# Malignant extra-adrenal pelvic paraganglioma in a paediatric patient

**DOI:** 10.3332/ecancer.2017.761

**Published:** 2017-08-23

**Authors:** Gabriel Cao, Julian Mendez, Daniel Navacchia

**Affiliations:** Anatomical Pathology Division, ‘Pedro De Elizalde’ Children’s Hospital, Montes de Oca Avenue 40, Buenos Aires C1270AAN, Argentina

**Keywords:** paraganglioma, extra-adrenal, malignancy, paediatrics

## Abstract

The extra-adrenal paraganglioma is a neoplasm originating in regional structures, uncommon in paediatrics. We report on a case of a 13-year-old patient who began with severe arterial hypertension, tachycardia, dilated cardiomyopathy and elevated levels of catecholamines in the blood and urine. The presence of a retrovesical pelvic mass in contact with the right vaginal dome was determined by imaging studies. A diagnosis of malignant extra-adrenal pelvic paraganglioma with lymph node metastases was reached through biopsy and the surgical resection of subsequent local recurrences. Paragangliomas are usually located in the paravertebral zones from the base of the skull to the retroperitoneum and are benign in 90% of cases. This kind of neoplasia is uncommon in paediatrics, especially those located in the pelvis. In cases of masses of a gynaecological origin, a differential diagnosis should be considered, and a histological and immunohistochemical study is essential in certifying the diagnosis.

## Introduction

Paraganglioma is a rare neuroendocrine neoplasia uncommon in the paediatric population originating in regional structures derived from neural crests. It is considered that between 10% and 20% of cases are diagnosed in the paediatric age group, with an estimated incidence of 0.011 for each 100,000 children under 18 years of age. Of these, approximately 12% are considered malignant [1–3]. Accordingly, the only widely accepted criterion is the presence of metastases in organs where normally there are no chromaffin cells, as is the case of lymph nodes [3, 4]. Extra-adrenal paragangliomas are usually diagnosed in the retroperitoneum, head, and neck. Those located in the abdomen and pelvis are associated with the sympathetic nervous system, with catecholamine hypersecretions being the cause of the signs and symptoms during the clinical presentation, as well as the possible effects of mass on neighbouring organs [5, 6]. The treatment of choice for this type of neoplasm is surgical resection and eventually, tumour embolisation prior to surgery or the administration of combined chemotherapy [3, 4].

## Case report

**Figure 1.**
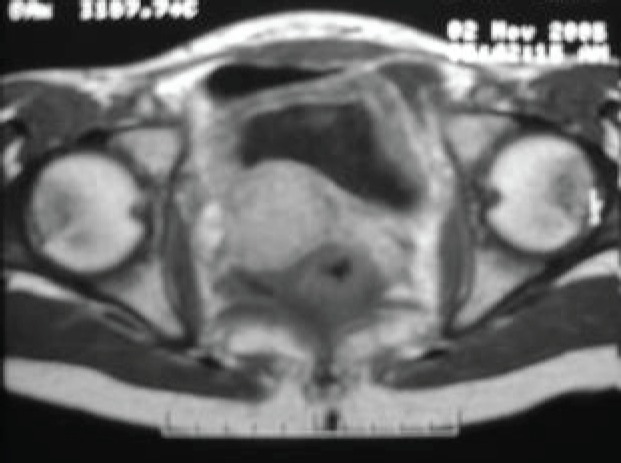
Magnetic resonance imaging.

**Figure 2.**
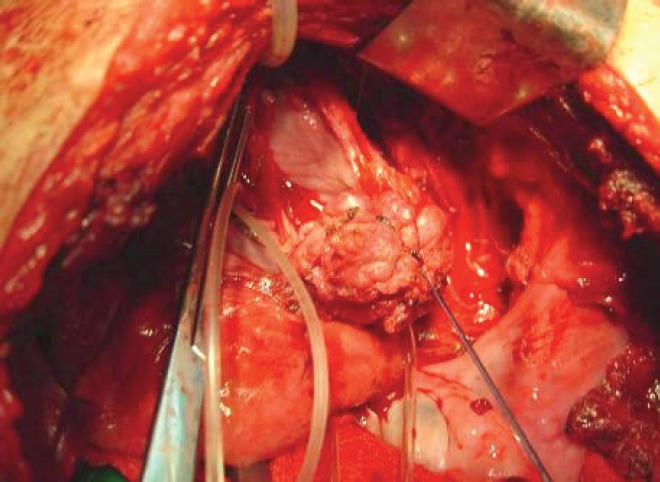
Dissection and resection of tumour formation.

**Figure 3.**
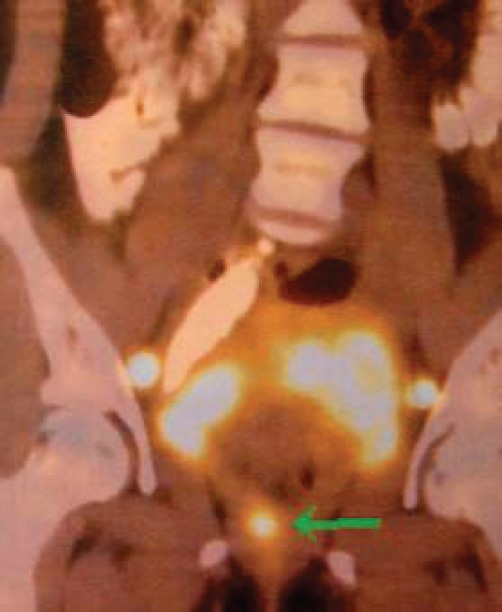
PET. Local recurrence.

**Figure 4.**
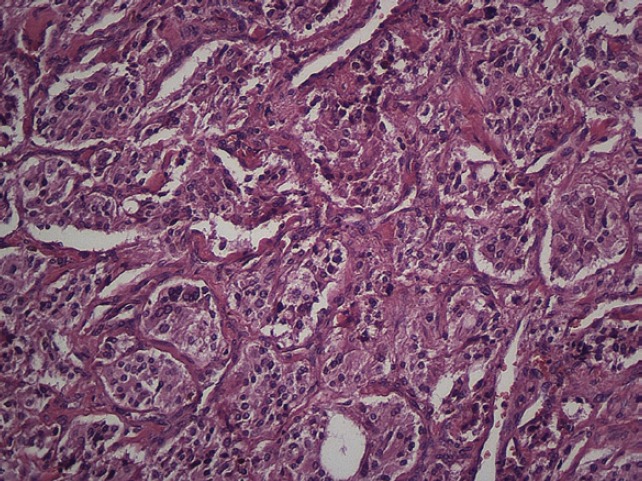
Microscopy. Haematoxylin and eosin. 20x.

**Figure 5.**
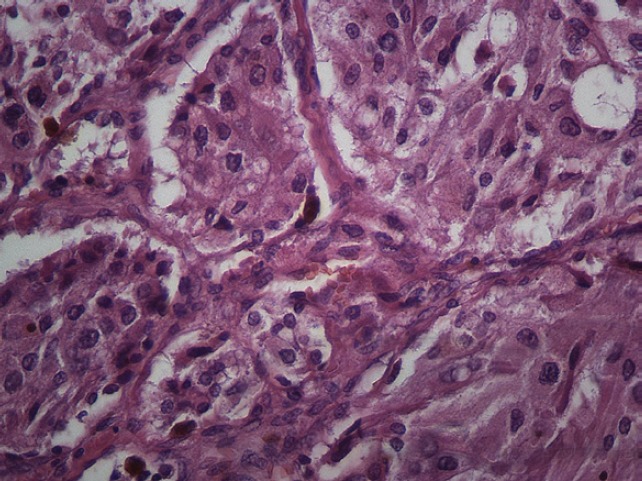
Microscopy. Haematoxylin and eosin. 40x.

**Figure 6.**
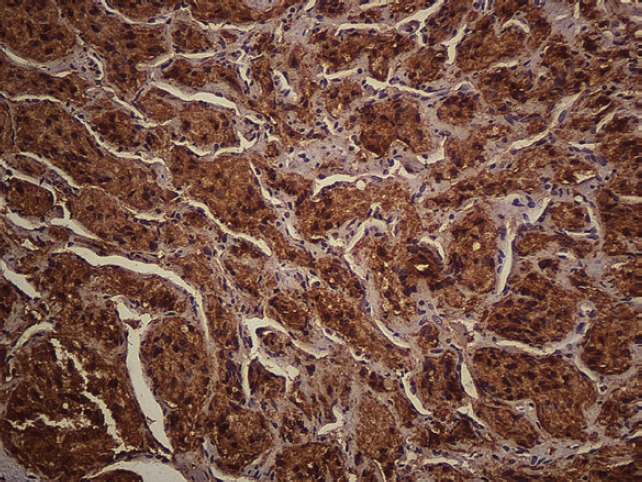
Immunostaining. Chromogranin-A. 20x.

**Figure 7.**
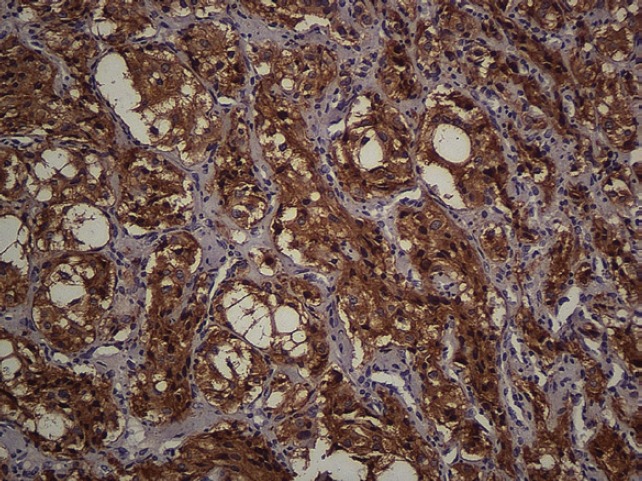
Immunostaining. Synaptophysin. 20x.

**Figure 8.**
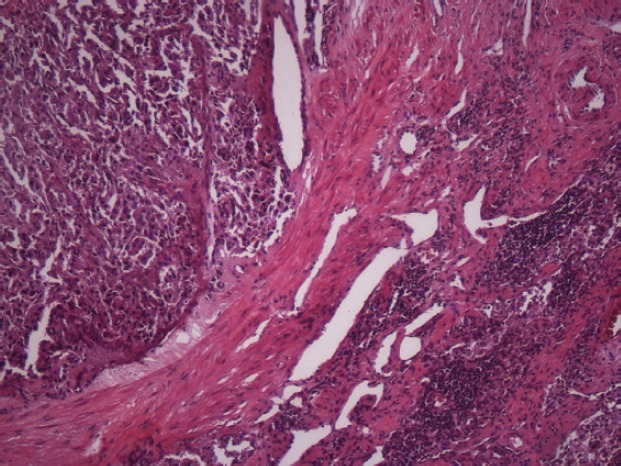
Haematoxylin and eosin. Nodal metastasis. 20x.

The subject is a 13-year-old female patient with a history of arterial hypertension, tachycardia, and cardiomegaly. In the clinical evaluation a palpable lump was found in the hypogastrium. Full laboratory tests were requested and the following relevant data were found: adrenaline and noradrenaline elevated in blood and urine. An ultrasound was carried out revealing an ovoid, heterogeneous solid mass of 4.4 × 3 cm in contact with the vagina and below the uterus, causing an effect of mass on the bladder. In order to determine the nature, characteristics, location, and relationships of this mass, magnetic resonance imaging (MRI) was requested and this reported an image of 4 × 6 × 4 cm, located in the retrovesical and right vaginal parauterine region in contact with the posteroinferior wall of the bladder, which it deforms (Figure 1). It was decided to perform a surgical biopsy of the lesion, obtaining the following anatomopathological results: paraganglioma. To assess functionality, the existence of lesions in other locations and the presence of metastases, a metamyodobenzylguanidine (MIBG) scan was requested, which was negative for neuroendocrine tumour metastasis. It was decided to perform surgical resection prior to pharmacological treatment. A laparotomy was performed, revealing a tumour in the retrovesical, parauterine, and right paravaginal region in contact with the vaginal vault and lower back part of the wall of the bladder. The lesion was dissected and resected respecting the vagina, uterus, and bladder (Figure 2). Coinciding with the surgical manipulation of the lesion, the patient presented fluctuation of arterial pressure that required pharmacological management. The anatomopathological study of both the biopsy and the resection revealed a proliferation of neoplastic cells that were predominantly present in solid nests separated by fibroconnective vascular stroma. The cells showed eosinophilic cytoplasm and mild nuclear pleomorphism (Figures 4 and 5). Immunostaining techniques were performed for chromogranin A and synaptophysin, both markers being positive (Figures 6 and 7). With these findings, the diagnosis of extra-adrenal pelvic paraganglioma was reached. The patient evolved for three years with local recurrences and metastases in intrapelvic lymph nodes due to the neoplasia described. These results were obtained due to the studies of positron emission tomography that revealed three pelvic hypermetabolic nodules (right side: 14.2 mm, left side: 14, 9 mm and 10.3 mm), with a hypermetabolic focus on the floor of the pelvis relative to the vagina, to the right of the midline, adjacent to the metallic suture clip (Figures 3 and 8).

## Discussion

Malignant extra-adrenal paraglangliomas are rare neoplasms, with less than 3% reported in the literature. Approximately 75% of the cases are sporadic and 25% are hereditary, associated with a family cancer syndrome such as multiple endocrine neoplasia type-2, Von Hippel–Landau syndrome and neurofibromatosis type 1. Paraganglioma is the term applied to tumours that originate in the autonomic nervous tissue regardless of location. They are usually located in the paravertebral area and with a thoracolumbar distribution, from the upper cervical region to the pelvis. Tumours of the head, neck, and anterior mediastinum are associated with the parasympathetic nervous system, whereas those of the posterior mediastinum, abdomen, and pelvis originate in the sympathetic nervous system [7]. The latter derive from the so-called organ of Zuckerkandl [8, 9]. Despite a widespread distribution in the body, it is infrequently found in locations such as the urinary bladder, urethra, renal pelvis, and ureter [10].

The majority of cases are diagnosed in the third to fifth decades of life, with paediatric cases being uncommon, and with those located in the pelvis being even less common. They usually present as pain and/or abdominal mass. Approximately 15% are asymptomatic and present as incidentalomas. They are frequently benign (90% of cases), while 10% are potentially malignant, as in the case presented. Clinical and imaging parameters are insufficient to predict the biological behaviour of these neoplasms and the presence of distant metastases is the only histopathological parameter of malignancy. The presence of a malignant paraganglioma in the paediatric age group makes it necessary to define the biochemical phenotype and family history, since it may be part of a syndromic condition, as in the case of mutations in the gene that codes for the enzyme succinate dehydrogenase B subunit SDHB), C (SDHC) or D (SDHD). It is considered that about 25% of the cases are carriers of mutations in the germinal line [11–13]. Primary treatment of these injuries consists of complete surgical resection with subsequent follow-up imaging studies and the determination of plasma and urinary catecholamines.

## Conclusion

Paragangliomas of the abdominopelvic cavity are neoplasias that are particularly uncommon in paediatric age groups. Clinical findings and imaging studies are variable and can even present as asymptomatic even when they reach a large size. It is important to note that in cases of pelvic locations in female patients a differential diagnosis should be carried out with masses of gynaecological origin, certifying the diagnosis with the surgical resection of the same and a subsequent histopathological and immunohistochemical examination.

## Conflicts of interest

The authors declare that they do not have any conflicts of interest

## Authors’ contributions

All authors participated in the search for bibliography and of the preparation of the manuscript. All authors approved the final version thereof.
